# Exploring women's fear of childbirth in a high maternal mortality setting on the Arabian Peninsula

**DOI:** 10.1017/gmh.2015.6

**Published:** 2015-05-30

**Authors:** Annica Kempe, Töres Theorell, Fatoom Noor-Aldin Alwazer, Samera Abdullah Taher, Kyllike Christensson

**Affiliations:** 1 Department of Public Health Sciences, Karolinska Institutet, Stockholm, Sweden; 2 Department of the Secretary-General, National Yemeni Midwives Association, Sana'a, Yemen; 3 Department of Family Planning, Ministry of Public Health & Population, Sana'a, Yemen; 4 Department of Women's and Children's Health, Karolinska Institutet, Stockholm, Sweden

**Keywords:** Fear of childbirth, girl child, maternal mortality, Millennium Development Goals, pregnancy, Yemen

## Abstract

**Background.:**

Few studies from low-income countries have addressed women's fear of childbirth (FOC) although likely to affect women during both pregnancy and childbirth. The aim of this study was to explore FOC in a high maternal mortality setting in the Arab region, Yemen.

**Methods.:**

A multi-stage (stratified–purposive–random) sampling process was used. We interviewed 220 women with childbirth experience in urban/rural Yemen. Answers to the question ‘Were you afraid of giving birth?’ were analyzed using qualitative content analysis.

**Results.:**

Women perceived childbirth as a place of danger. Fear of death and childbirth complications stemming from previous traumatic childbirth and traumatic experience in the community was rampant. Husbands’ and in-laws’ disappointment in a girl infant constituted a strong sociocultural component of FOC. Women's perception of living in tension ‘between worlds’ of tradition and modernity reinforced fear of institutional childbirth. Women without FOC gave reasons of faith, social belonging and trust in either traditional or modern childbirth practice, past positive experience of childbirth and the desire for social status associated with children.

**Conclusions.:**

The numerous maternal and infant deaths have a strong impact on women's FOC. Antenatal care has an important role in reducing fear including that of institutional childbirth and in strengthening a couple in welcoming a female infant. Staff should be sensitized to the fears of both husband and wife and women be allowed support during childbirth. Within the scope of the Millennium Development Goals and strengthening of reproductive mental health programs, FOC urgently needs to be addressed.

## Introduction

Few studies have addressed women's fear of childbirth (FOC) in low-income or high maternal mortality settings. Though maternal mortality still lies at the heart of maternal health indicators, there is a growing concern about the impact of mental and behavioral health and emotional wellbeing on maternal health outcomes and the growth and development of children (World Health Organization, WHO & United Nations Population Fund, UNFPA, 2008). WHO (2014) is proposing a stronger focus on mental health for an integrated delivery of services for maternal and child health (MCH). Globally, an estimated 287 000 women died during pregnancy or childbirth in 2010 and for every woman who died, some 20 others faced serious or long-lasting consequences (WHO, 2012). For 1 million infants, their day of birth was also their day of death, and close to 2 million newborns died in the first week of life (United Nations Children's Fund, UNICEF, 2014). Facility-based childbirth remains low across regions (UNICEF, 2014). To gain knowledge of factors and obstacles, which influence women and their thoughts, emotions and wellbeing during pregnancy and childbirth is important for the accomplishment of Millennium Development Goal (MDG) 5, which focuses the improvement of maternal health (WHO, 2000). Such knowledge would help staff to better interact with women clients and thus increase the proportion of births attended by skilled health personnel.

Childbirth fear has been associated with adverse maternal outcomes, including childbirth complications, high rates of caesarian, poor postpartum mental health and impaired maternal–infant interaction (Ryding *et al*., 1998, Söderquist *et al*., 2009, Rouhe *et al*., 2011, Adams *et al*., 2012, Storksen *et al*., 2012). In Africa, studies using a broad approach to antenatal stressors found pregnancy in general to be a time of vulnerability and fear (Hanlon *et al.*, 2009, 2010, Dako-Gyeke *et al.,* 2013, Stewart *et al*., 2014). Studies of FOC have been conducted in Asia (Tsui *et al*., 2006, Sercekus & Okumus, 2009, Matinnia *et al*., 2014, Takegata *et al*., 2014), Australia (Fenwick *et al*., 2009, Haines *et al*., 2011, Toohill *et al*., 2014), Canada (Hall *et al*., 2009), Scandinavia (Areskog *et al*., 1981, Zar *et al*., 2001, Kjaergaard *et al*., 2008, Nieminen *et al*., 2009, Rouhe *et al.,* 2009, Salomonsson *et al*., 2013), Western (Johnson & Slade, 2002, Sluijs *et al*., 2012, Ayers, 2014) and Central Europe (Jokic-Begic *et al*., 2014, Dweik *et al*., 2014). A European study of six countries found that FOC was reported by 11% of all women although the content of it varied between countries (Lukasse *et al*., 2014).

Yemen is one of the ten countries chosen for the United Nations (UN) Millennium Project. Yemen ranks low in the Human Development Index and last in the Gender Inequality Index (UN Development Programme, UNDP, 2014). The maternal mortality ratio (MMR) in Yemen is estimated to be 200 maternal deaths per 100 000 live births (UNDP, 2014). Skilled attendance at birth remains low at 12% in rural and 34% in urban areas (Al Serouri *et al*., 2012). Antenatal coverage is estimated to be approximately 47% for one visit; only 11% of women attend for four visits as recommended by the WHO. Midwives practice at all levels of the health care system: in hospitals, health clinics/units and in the community.

## Aim

The aim of the study was to explore women's FOC in a high maternal mortality setting.

## Methods

### Study setting, participants and data collection

This study is part of a larger research project, aiming to gain insight into the experience of Yemeni women of modern and traditional care during childbirth, conducted in the governorates of Aden, Lahej, Hadramout, Taiz and Hodeidah. A multistage (stratified–purposive–random) sampling process was used to select a total of 220 women, 44 from each governorate. The study group was defined as the female head of each identified household with experience of childbirth. A detailed account of the study design has been published (Kempe *et al*., 2010).

Study participants were interviewed using a questionnaire with closed and open-ended questions covering their most recent pregnancy and childbirth, postpartum period, women's empowerment and sociodemographic information. A pre-test of the questionnaire was carried out in Taiz. The question about women's FOC was formulated as: ‘Were you afraid of giving birth? Yes/no. Please explain’, allowing maximum freedom for respondents to give voice to their most urgent concerns. Answers were manually recorded in notes. The question was analyzed using qualitative content analysis (Graneheim & Lundman, 2004). Answers were first categorized as affirmative or non-affirmative to FOC. After repeated readings of all answers, themes emerged in the two groups. Categories were identified under each theme, and quotes were selected that typified most women's responses.

A multidisciplinary team conducting the field study comprised the principal investigator (PI) with a background in social anthropology, two Yemeni nurse-midwives and two medical doctors, one of whom an obstetrician/gynecologist and one a pediatrician, and one Sudanese nurse-midwife. The purpose of the study, emphasized by all, was to give voice to Yemeni women in childbirth and present a local perspective.

### Trustworthiness

Special measures were taken to ensure trustworthiness of the data. Interviews proceeded at the pace set by each individual woman, thus were sometimes interrupted for cooking, gathering a flock of sheep from a nearby mountain or hanging wash to dry on the roof. Interviews lasted for 1–3 h. Some of the women's husbands or older female relatives would sometimes sit and listen during the initial phase of the interview, which could inhibit the women to speak freely. It was decided that in such cases interviewers had to return a second time to secure privacy. All performed interviews were discussed the same evening among team members. During the process of analysis, in-depth discussion of findings took place between the PI and one more member of the team.

## Results

The majority of women in the study group (*n*: 158, 72%) had experienced FOC and a minority (*n*: 62, 28%) had not. The themes and categories associated with fear and non-FOC are illustrated in Table 1. Within answers, categories are not mutually exclusive.

**Table 1.** Women's FOC and non-FOC in a high maternal mortality setting on the Arabian Peninsula (n: 220)

**Themes of fear**	**Categories**	**From quotations**
Childbirth as a place of danger	Fear for self and infant	‘It's a fright – a place between life and death’
	Past traumatic childbirth	‘When I remember I wish to never give birth again’
	Too far to walk	‘No medical care and no roads’
Maintaining control in the familiar	Fearing the hospital	‘Only God can save my baby and myself’
	Fearing institutional staff	‘I felt helpless’
	Fearing modern delivery practice	‘I didn't know anyone around me’
Living in the tension of transition	Conflicting beliefs, conflicting knowledge	‘I was afraid because my husband was afraid’
The subordinated mother	Fearing the girl infant, needing the boy	‘I say: please be a boy’
	Children as a means to status	‘If there were children to buy I would have given all my gold’
**Themes of non-fear**	**Categories**	**From quotations**
Childbirth as fate	God's will	‘No one dies before his or her time’
Childbirth as trustworthy	The power of a positive past	‘I have been pregnant twenty-four times’
	Giving birth as a socially privileged woman	‘I was in the right place with the right person’
	Trusting the care of traditional childbirth	‘The women around me hold me tight’
The desire for children	Children as a gift of God	‘Birth brings a good present.’
	Children as a means to status	‘Men like children, so I'm never afraid’
Non-fear as childbirth tradition^a^	Trusting in self	‘God gave me the courage’
	Perceiving of support by a higher benevolence	‘I don't know the word afraid at all’
	Boys and girls are equal	‘All are children of God’

a This childbirth tradition is prevalent in the Hadramout governorate of Southeast Yemen and is especially strong among the Nomad population.

**Fig. 1.**
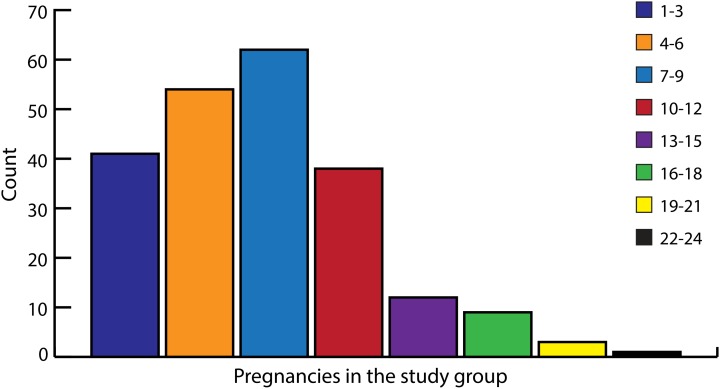
Pregnancies in the study group (*n*: 220). Cumulative number of pregnancies *n*: 1653, range 1–24.

**Fig. 2.**
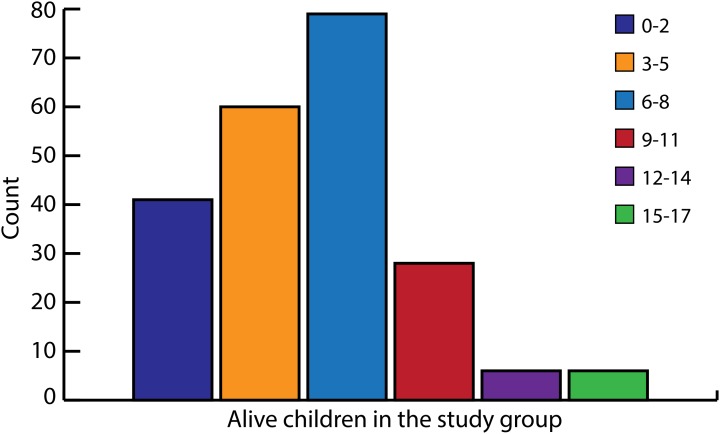
Alive children in the study group (*n*: 220). Total number of living children *n*: 1273, range 0–16.

Twelve women had given birth once. The remaining 208 women had given birth between two and 16 times. The cumulative number of pregnancies in the study group as were reported by women are shown in Figure 1. Figure 2 shows the total number of living children at the time of interview. The average number of pregnancies among women was 7.6 and the number of living children 5.8.

Table 2 presents the social and demographic characteristics of women in the study and information regarding health care seeking during pregnancy and childbirth.

**Table 2.** Background data of women in the study group (n: 220)

**Social and demographic factors**		
	Number	(%)
Age		
<25 years	60	27
25–35 years	114	52
>35 years	46	21
Place of residence		
Urban	112	51
Rural	108	49
Dependents		
2–5	47	21
6–9	64	29
10–13	69	32
>14	40	18
Distance by walking to location of childbirth		
None (home birth)	153	69
<half an hour	19	9
Half an hour–1 h	7	3
1–2 h	1	0.5
>2 h	40	18
*Literacy and education*		
Woman's literacy and education		
Illiterate	154	70
Reads Quran	6	3
Primary school	39	17
Intermediary school	6	3
Secondary school	13	6
Higher education	2	1
Husband's literacy and education		
Illiterate	85	38
Reads Quran	8	4
Primary school	40	18
Intermediary school	22	10
Secondary school	30	14
Higher education	35	16
*Occupation and income*		
Woman's wage employment outside home during the last 3 months	17	8
Occupational status of husband		
Farmer/laborer	98	45
Government employee	122	55
Other family income (land, cattle, income from abroad)	49	22
Woman's life experience of loss of child	148	67
1–3 children	106	48
4–6 children	34	15
7–9 children	8	4
**Pregnancy care**		
	Number	(%)
Informal sector: by a traditional birth attendant (TBA) with a traditional vocational training and/or by family members/women in surrounding	68	31
Formal ANC sector: by a formally trained TBA^a^, Female primary health care provider, midwife or medical doctor	152	69
ANC attendance by type of care provider (*n*: 152)		
TBA, formally trained	3	2
Female primary health care provider	26	17
Community midwife	11	7
Registered nurse midwife	72	48
Medical doctor	40	26
Number of ANC visits (*n*: 152)		
1–3	38	25
4–6	73	48
7–9	41	27
**Childbirth care**		
Informal sector: non-assisted childbirth^b^ or attended by a TBA with a traditional vocational training	101	46
Formal sector: by a formally trained TBA, female primary health care provider, midwife or medical doctor	119	54
Childbirth attendance by:		
No one	30	14
TBA with a traditional vocational training	71	32
TBA, formally trained	10	5
Female primary health care provider	27	12
Community midwife	16	7
Registered nurse midwife	29	13
Medical doctor	37	17
Location of childbirth		
Home	152	69
Clinic/health unit	13	6
Hospital	55	25

a TBA's undergo a traditional vocational training together with an older TBA or have received formal training of 1–8 weeks at the local health center or health sub-center.

b Non-assisted childbirth is tradition in the Hadramout governorate of Southeast Yemen.

### Women with FOC

Four themes emerged from the analysis: (i) ‘childbirth as a place of danger’ including three categories ‘fear for self and newborn’, ‘past traumatic childbirth’ and ‘too far to walk’; (ii) ‘maintaining control in the familiar’ including the categories ‘fearing the hospital’, ‘fearing staff’ and ‘fearing modern delivery practice’; (iii) ‘living in the tension of transition’ including the category ‘conflicting beliefs, conflicting knowledge’ and (iv) ‘the subordinated mother’ including two categories ‘fearing the girl infant, needing the boy’ and ‘children as a means to status’.

### Childbirth as a place of danger

This theme illustrates the perception of nearly all women with FOC. Fear stems from the reality in which women live and reflects the situation, both in rural and urban Yemen, of the high maternal, perinatal and neonatal mortality.

#### Fear for self and newborn

‘*It's a fright – a place between life and death.*’ *(Para 4, 3 living children, age 30 years, urban)*

‘*I'm afraid of death.*’ *(Para 9, age unknown, rural)*

‘*Very dangerous position for every woman.*’ *(Para 7, age 25 years, rural)*

‘*Birth is suffering – one doesn't believe that one can deliver safely.*’ *(Para 12, 8 living children, age unknown, age of eldest son known to be 38 years, rural).*

A large majority of women with FOC described childbirth as a matter of life and death. Many had intimate experience of someone close dying in childbirth and told of sisters, neighbors and friends. Some women had lost their mothers in childbirth, either at their own birth or that of a brother or sister. As a consequence, women feared both pregnancy and childbirth.

‘*I'm afraid because my sister died during childbirth last year.*’ *(Para 8, age 22 years, rural)*

‘*My mother died when she was carrying a baby.*’ *(Para 6, 2 living children, age 34 years, urban)*

‘*Because pregnancy means that you may die, I hope I will never again become pregnant.*’ *(Para 4, 3 living children, age 22 years, urban).*

Three women had experienced the death of their infants at the previous childbirth and many had a history of losing infants two, three or many more times. During the pregnancy, such fears were the subject of direct communication with the unborn.

‘*I asked the baby to be alive.*’ *(Para 9, 4 living children, rural)*

#### Past traumatic childbirth

‘*Because of a bad history I'm afraid. The first baby was stillborn, the second a caesarian operation, also the third baby was a caesarian operation.*’ *(Para 3, 2 living children, age 25 years, urban)*

Around a third of the women related fear to past trauma during childbirth. Women described many kinds of problems, of which hemorrhage and prolonged labor were the most common. Hemmorhage had occurred to women sometimes before birth, sometimes after. The dangers connected with hemmorhage after childbirth was what women feared the most.

‘*Because of hemmorhage and nearly dying before I keep worrying all the time of giving birth.*’ *(Para 10, 6 living children, age 25 years, rural)*

Memories of traumatic circumstances connected with prolonged labor were often recalled, such as the pain of a forceps delivery, the loneliness in a hospital after prolonged labor and the difficulty to ask for help among strangers. Other childbirth complications, which raised fear in women were past incidences of retained placenta, difficult positions of the baby as breech or transverse lying, vaginal infections, fainting, illness and vaginal tears. Pain once experienced was often recalled and contributed greatly to women's fear.

‘*When I remember the tear I wish to never give birth again.*’ *(Para 5, age 34 years, urban)*

‘*Giving birth is painful, if the baby or the placenta remain, it means I will die.*’ *(Para 2, pregnant, age 23 years, rural)*

Rural women often referred to themselves as a collective when telling about their difficult plight.

‘*Very frightened from pain and complications, but God help us.*’ *(Para 10, pregnant, 7 living children, age 30 years, rural)*

#### Too far to walk

Reaching the hospital in times of emergency was often very difficult, sometimes impossible for rural women. A small number of women gave distance to delivery care as a reason for their FOC. These women were always afraid, since there is no car in the village. The cost for renting a car to go to the hospital was mentioned by some as the direct reason for their fear of birth complications.

‘*I'm here in the mountains, no medical care and no roads. If something bad happens, it's difficult to go to hospital.*’ *(Para 4, 2 living children, age 17 years, rural)*

### Maintaining control in the familiar

During childbirth, familiar circumstances and habits were strongly preferred and took on great importance for women, especially rural women, who for the most part expressed resistance to change. Around a third of the women with FOC expressed fear of the hospital and smaller groups voiced specific fears of staff, modern delivery practice or both.

‘*It's good to give birth in your own house and I never went out of this place since I married. I leave only when I have to bring wood and water.*’ *(Para 3, 2 living children, age 22 years, rural)*

‘*I deliver all my children in the same room at home.*’ *(Para 13, 12 living children, own estimated age 50 years, urban)*

Fear of the hospital, of institutional staff and of modern delivery practice constituted overlapping yet different aspects of women's FOC, where one kind of fear sometimes concealed another.

#### Fearing the hospital

Rural and to an extent also urban women expressed fear of the hospital, which was often seen as the symbol of everything women feared around childbirth. Fear of birth complications was frequently mentioned as an indirect fear of the even greater fear of having to go to the hospital.

‘*I was afraid of any complications which might take me to the hospital, only God can save my baby and myself.*’ *(Para 10, 5 living children, age 35 years, rural)*

Traumatic memories of staying in the hospital were recalled causing fear of returning again to the place of trauma.

‘*I'm afraid of a hospital birth and of bleeding. I suffered from both during my first pregnancy and I'm still afraid.*’ *(Para 14, 7 living children, age 30 years, urban)*

‘*So frightened because during my last delivery I had retained placenta and they removed it in the hospital. I'm afraid it will be the same*’ *(Para 3, pregnant, age 25 years, urban)*

Some women did not desire any more children for the reason of risking a hospital birth. These were women with prior experience of hospital birth and without such experience.

*‘I only pray to God to never give me more children, and if I become pregnant, I will die in my house and not go to hospital.*’ *(Para 12, twins 3 times, 9 living children, age 29 years, urban)*

Fear of pain was sometimes voiced in conjunction with entering unknown territory.

‘*I'm afraid of severe pain and the new people and the new place.*’ *(Para 4, age 27 years, rural)*

#### Fearing staff

Women feared the loneliness of a hospital birth. Generally, women expressed feelings of anxiety to be in the hands of staff they experienced as only remotely present with them during childbirth. That staff refused women the company they needed to feel safe was a cause for long-lasting negative emotion. The denial during childbirth of what women wanted for their comfort was another expression of unhappy situations with staff.

‘*I asked for my mother to enter the labour ward, but they refused. I felt helpless. I asked for water, my husband brought it but they didn't give it to me to drink.*’ *(Para 3, age 23 years, urban)*

#### Fearing modern delivery practice

Fear of delivery practice was given as a reason for FOC in a small number of cases. In the hospitals and some clinics, women were expected to give birth in the lying supine position, which according to women would make the baby fall sleep. Episiotomies were sometimes more painful than childbirth itself.

‘*I wished that my delivery would not go on and everything would stop.*’ *(Para 2, age 29 years, urban)*

Regulations against allowing support during childbirth by an additional person besides the midwife or doctor were especially difficult for rural women, who often arrived at the hospital already traumatized. Such separation from loved ones had, for some women, negatively influenced the progress of labour.

‘*Because my mother was not there, I suffered prolonged labor.*’ *(Para 4, 2 living children, age 29 years, urban)*

For even more women, the anxiety and loneliness they experienced rendered childbirth an unhappy experience.

‘*This was the first time I gave birth without my mother's presence. I felt lonely.*’ *(Para 10, 7 alive children, age 36 years, urban)*

### Living in the tension of transition

#### Conflicting beliefs, conflicting knowledge

The husband's FOC in a few cases became the source of women's fear. This was more common among urban women, whose husbands had acquired knowledge about the risks of childbirth. Husbands were by far more educated than their wives and in many cases had personal connections with the doctors in the hospital. As a consequence, husbands often lived in a different world from women – a world where FOC is called for. Women subjected to the authority of the husband took on his fear, sometimes resulting in confusing situations.

‘*I was afraid this time very much because of my husband. He was very afraid, and whenever I saw him leave the house, I thought something might happen to me.*’ *(Para 2, 1 living child, age 14 years, urban)*

### The subordinated mother

This theme conveys women's experience of being subordinated to the expectations of family and society. A majority of women, depending on reproductive history, alternately voiced their fear about the gender of the infant, about having children, many children or all.

#### Fearing the girl infant, needing the boy

To become a mother of a male infant was a predominant desire and few exceptions to this were found in the study population. Many gave evidence of a constant anxiety.

‘*I was thinking all the time about having a baby girl again, and I felt all the time sad.*’ *(Para 5, age 27 years, rural)*

‘*I touch my abdomen and talk to myself like a madwoman, and I say: please be a boy, so that your father will be happy and I will be called a mother of boys.*’ *(Para 11, 8 living children, age 27 years, rural)*

Women recalled the stress from childbirth and how the pressure felt from the husband made them sick and unable to proceed with labor.

‘*I had nine daughters, my husband wants a son. I was so frightened it's a girl that I started a fever and was shivering from fright.*’ *(Para 10, pregnant, age 33 years, urban)*

‘*My husband is only waiting to hear the good news that the baby is a boy, he never thinks of me, only the gender of the baby, and I feel very badly.*’ *(Para 4, 3 living children, age 22 years, rural)*

Among the few exceptions to desiring a boy, one respondent communicated to a much longed for girl infant her mother's acute need for support.

‘*I always talk to myself and the unborn baby and I tell her that I'm alone and I need her to come out as an alive girl, so she may help me.*’ *(Para 4, 3 living children, age 23 years, rural)*

#### Children as a means to status

To be a mother of many children was desirable particularly among the rural half of the study population. Status within family and in the community followed and this had led to a situation with women living in constant fear of child loss before, during and after childbirth. A respondent explained how infertility had caused anxiety throughout her adolescence.

‘*I married at the age of 12 and after nine years I became pregnant. If there were children to buy I would have given all my gold to have one, male or female. I experienced a lot of problems for children. My husband married three times and divorced each co-wife after two years. I thank a merciful God that I'm now living happily with children.*’ *(Para 10, 8 living children, age 39 years, urban)*

### Women without FOC

Four themes emerged from the analysis in the group of women without FOC: (i) ‘childbirth as fate’ including one category ‘God's will’; (ii) ‘childbirth as trustworthy’ including three categories ‘the power of a positive past’, ‘giving birth as a socially privileged woman’ and ‘trusting the care of traditional childbirth; (iii) ‘the desire for children’ including two categories ‘children as a gift of God’ and ‘children as a means to status’; and (iv) ‘non-fear as childbirth tradition’ including the categories ‘trusting in self’, ‘perceiving of support by a higher benevolence’ and ‘boys and girls are equal’.

### Childbirth as fate

#### ‘God's will’

Both rural and urban women expressed the belief that childbirth is of God's will and that they therefore will be given the courage to endure it. This was true for about a quarter of women who lacked FOC. Why should they be afraid, women asked, when they know that God's will prevails?

‘*I think our lives are in God's hands and God only can make childbirth easy. I believe that no one dies before his or her time.*’ *(Para 10, 8 living children, age 39 years, urban)*

### Childbirth as trustworthy

The perception of childbirth as trustworthy was found among both rural and urban women. Around half of the women who lacked FOC expressed such trust. Certain situations and circumstances allowed these women to be less afraid during childbirth. A positive previous experience of childbirth was referred to by a small group of women. Women's trust in staff and other care providers was a unifying factor in experiencing childbirth with less or no fear, found among women in both the modern and the traditional sectors.

#### The power of a positive past

Despite multiple childbirths, some women did not suffer ill health and stated their implicit trust in childbirth.

‘*How would I be afraid, I have been pregnant twenty-four times and I'm still in good health.*’ *(Para 24, 15 living children, age 40 years, rural)*

Childbirth was seen as a normal event, sometimes even regardless of its outcome. The lack of fear among these women was often explained by their recall of the positive experience of persons close.

‘*I was afraid during the first pregnancy, but this time I leave everything to God. He is the only one who can help and I think about my mother who gave birth nineteen times and who is still in good health.*’ *(Para 3, 2 living children, age 19 years, rural)*

#### Giving birth as a socially privileged woman

Socially privileged urban women with institutional childbirth experience expressed their trust in the midwives and medical doctors as the primary reason for their lack of FOC. Personal connections with staff and previous childbirth in the same place with the same midwife created the sense of safety.

‘*I felt I was in the right place and with the right person to take care of me.*’ *(Para 1, age 20 years, urban)*

But also among the more privileged women, childbirth was mostly seen as an event to be supported by others. For women with a home birth and in the few cases of institutional childbirth where support had been allowed, the presence of the husband, mother or mother-in-law together with a trained midwife was the key to the lack of fear.

#### Trusting the care of traditional childbirth

Women in rural areas who lacked FOC also stated their full confidence in the midwife – most often a traditional midwife – and in God, the great helper. Rural areas were sometimes very inaccessible with steep hills and dangerous terrain to travel, especially at night. When the midwife finally arrived, fear dissipated. The calming influence of the traditional midwife on both rural and urban women was explained by the faith of these midwives in a natural course of childbirth. Other women's comforting acts during childbirth was something else women had learnt to depend on. Childbirth, particularly in the rural areas, is by tradition a shared event.

‘*The women around me, they do what I ask them for, they massage my back, put oil on my hair and hold me tight.*’ *(Para 7, 1 living child, age* ‘*maybe 20–30 years*‘, *rural)*

### The desire for children

#### Children as a gift of God

Rural women and to an extent also urban women viewed children as a gift from God.

‘*Birth means severe pain for a short period but brings a good present from God: a new baby.*’ *(Para 10, 8 living children, age 39 years, urban)*

#### Children as a means to status

Rural women in particular sometimes referred to the state of motherhood itself as a reason not to be afraid of childbirth. Fulfilling a woman's duty to become a mother lessened the fear. The desired status that many children give was often emphasized.

‘*I like children. If I become afraid, how can I bring children? And you know, men like children, so I'm never afraid.*’ *(Para 11, 8 living children, age 27 years, rural)*

### Non-fear as childbirth tradition

Women residing in the Hadramout governorate constituted one fifth of the study population. Among Hadramout women expressions of non-FOC were the most common. Hadramout women described a contrasting outlook on childbirth. Childbirth is by tradition unassisted. Girls in this part of the country are socialized from early life into a cosmology of non-fear and have different role models with regards to pregnancy, childbirth and motherhood. Mothers and grandmothers are instrumental in transmitting the knowledge, attitudes and practices of this childbirth tradition. The Hadramout governorate is also the home of a large segment of Yemen's Nomad population, with a distinct childbirth tradition emphasizing non-fear, which stems from the matriarchal roots of that society.

#### Trusting in self

‘*God gave me the courage to perform the delivery by myself*’ *(Para 9, 7 living children, age unknown, rural)*

Hadramout women claimed in a majority of cases that they could always count on accessing the strength they need during childbirth, as they are one with God. During childbirth, they are directed in what to do and to trust in their own ability to handle any demands put on them.

#### Perceiving of support by a higher benevolence

‘*When you give birth alone, you feel that God is with you.*’ *(Para 8, 7 living children, age 30 years, rural)*

Women explained that they could always count on receiving the support they need from the benevolent powers of the universe, during childbirth as in life itself. The small group of Nomad women interviewed in particular expressed that their lack of FOC was a natural consequence of fully trusting in the power of God in all spheres of life.

‘*I leave my life to God and I don't know the word afraid at all. And why should I be afraid of God? I didn't make him angry.*’ *(Para 6, age 26 years, rural, Nomad woman)*

#### Boys and girls are equal

Nomad women in some cases emphasized the equal value of boys and girls. The birth of a daughter or a son was as valuable and this, women underscored, is a viewpoint embedded deep in the fabric of their society.

‘*All are children of God.*’ *(Para 6, 5 living children, age 18 years, rural, Nomad woman)*

## Discussion

This study of women's FOC in a high maternal mortality setting shows that a vast majority of women experienced substantial fear. Psychological as well as sociocultural factors were shown to be deeply at play. FOC was perceived on a personal as well as on a community level. It showed intricate and complex relationship with a woman's status within her family and in society but also with maternal and child healthcare.

To our knowledge, this is the first study to directly address FOC in a low income or high maternal mortality setting. The strength of the study is that women were approached for interview at home, thus given the opportunity to voice their concerns. Local team members had a long professional experience of working closely with women in rural/urban Yemen. Recall bias might have constituted a limitation of the study. Research however shows that women are able to recall their memory and impressions of childbirth accurately for a long time (Waldenström & Schytt, 2009).

The burden of childbirth complications caused psychological distress in the every-day lives of women. In Yemen as in other low-income settings, childbirth is by tradition shared among women in the community (Kempe *et al*., 2011, 2013, Kyomuhendo, 2003, Kaphle *et al*., 2013). Most women without a personal experience of traumatic childbirth knew and were influenced by other women with such experience. Little research exists about the role of community in the development of FOC. A study about maternal fear associated with pregnancy and childbirth in Hong-Kong Chinese women identified the main factor causing fear as ‘negative stories’ of women in surrounding (Tsui *et al.,* 2006). In Australia, Fisher *et al*. (2006) explored the role of social context in connection with FOC. Prospective fear was seen in this study as both social and personal and retrospective fear exclusively as personal.

Previous childbirth trauma was common among women with FOC, confirming findings from Western settings (Rouhe *et al*., 2009, Nilsson *et al*., 2010, Haines *et al*., 2011, Storksen *et al*., 2013). The loss of infants during pregnancy, childbirth and the postpartum period was deeply traumatizing to women, as in Western society (Trulsson & Rådestad, 2004), and solace in the community the only relief. Women with a previous pregnancy loss are more likely to experience sadness, low mood and excessive worry also in a subsequent pregnancy (Chojenta *et al*., 2014) and more vulnerable to post-traumatic stress disorder (PTSD), especially when conception occurs close to the loss (Turton *et al.*, 2001).

It is important to remember, in this cultural setting, that most women were married young, or very young, as tradition calls for. The youngest interviewee was only 14 years old. Regardless of age during the time of interview, the base of FOC may thus be the childbirth experience of someone who was no more than a child at first birth. According to UNICEF and Yemeni government data (2008) half of girls are married before the age of 18 and 14% below 15 years. With little education and power in their marriage, girls in Pakistan have been shown to have little chance to control their fertility, which increased their risk of reproductive health problems (Hamid *et al*., 2010). Early marriage has been linked with interpersonal violence in Vietnam (Hong *et al.,* 2014) and a history of early abuse to severe FOC among primiparas in Norway (Lukasse *et al*., 2010). In Yemen early marriage has received ample attention lately not least because children themselves have spoken out against it, and measures are being taken on part of the Human Rights Ministry to legalize marriage at the age of 18 years.

The wider sociocultural context provided fertile ground for FOC in that it gave rise to anxiety about motherhood, forcing women's thoughts to be preoccupied with the need to give birth to a male infant. The emotional distress caused to women by gender preference and its adverse effects on women's wellbeing postpartum is known from a range of settings in the Middle East and Asia (Rodrigues *et al*., 2003, Patel & Prince, 2006, El-Gilany & Shady 2007, Xie *et al*., 2009, Mohammad *et al*., 2011) and is interpreted from the context of social adversity, poor marital relationships and cultural attitudes toward gender rather than a biomedical psychiatric category. A systematic review on pre- and postnatal psychological wellbeing comprising eight African countries, conducted by Sawyer *et al*. (2010), showed depression to be the most commonly assessed disorder. Depression was related to the lack of support of women and marital/family conflict. Alipour *et al*. (2012) studied the relationship between anxiety during pregnancy and postpartum depression in Iran, concluding that prenatal anxiety could be an important factor in postpartum depression and that the focus needs to be changed from postpartum depression to prenatal anxiety.

An important aspect of women's fear concerned institutional childbirth and delivery practice. A study in Yemen's capital Sana'a (Basaleem, 2012) showed that 47% of births were attended at home and 36% of these births only by a skilled birth attendant. In their review of facilitators and barriers to facility-based delivery in low- and middle-income countries Bohren *et al*. (2014) concluded that due to the abundant experience of disrespectful and abusive obstetric care, future research should focus on achieving respectful, non-abusive and high-quality obstetric care for all women. Improving perinatal care is likely to be an important component of interventions to tackle antenatal mental ill health (Hanlon *et al.*, 2009).

Maternity caregivers in our study were identified as both a cause of FOC and a pivotal mediating factor in reducing it. Women lacking FOC were found across the institutional and home sector. Trust in staff and other care providers was the unifying factor of experiencing childbirth with less fear. Publications from other settings have described the ability of staff to reduce FOC (Hildingsson *et al.*, 2011, Salomonsson, 2012) and women in Yemen seem to differ little from women elsewhere. In Yemen as in similar low-income country settings however, restrictions of time and resources to secure a woman's needs during childbirth are often more acute. A Swedish study (Swahnberg & Wijma, 2011) examining staff awareness of abuse in healthcare found that staff awareness varies according to context and possibilities to act.

The integration of practice from the traditional childbirth sector (Kempe *et al*., 2010, 2011, 2013, Dako-Gyeke *et al*., 2013) would be important in local intervention to reduce FOC and to increase utilization of skilled care. When women have been expected to conform to a different system of childbirth practice, the unwillingness to change their ways, stated clearly in women's comments, should be taken to heart. Low trust in the health care system has been related to psychological distress (Ahnquist *et al*., 2010). In a recent European study (Lukasse *et al.,* 2015), the reporting of abuse in health care among women attending routine antenatal care (ANC) was shown to be closely associated with FOC especially for nulliparous women. Women who reported such abuse more often suffered from other forms of abuse, economic hardship and negative life events as well as from a lack of social support, symptoms of post-traumatic stress and depression. Counseling with groups of women would be interesting and has been highly appreciated in Yemen (Kempe *et al*., 1994). Toohill *et al.* (2014) found that trained midwives are able to effectively reduce high FOC levels and increase childbirth confidence in women from mid to late pregnancy. Listening and responding to women's feelings and asking explicitly about women's fears were important. In ANC culturally based arguments could be used to strengthen both husband and wife in welcoming a female infant. In Yemen girls are known to help their parents more than the boys. Parents could be encouraged to think that a girl will study, find work and have her own income. Schools could be used for health education with children. In Aden (Southern Yemen) some decades ago, health awareness was taught from primary school. Step by step, boys and girls were led to think about the procrastination of marriage and the value of family planning. Simlar methods could be used to promote the equal value of the female. Women were once queens in Yemen.

What were some of the factors of resilience among the close to 30% of women who were not afraid? Women's faith in God, trust in childbirth and in the care provided, the overriding joy to become a mother and accompanying status in the society were all contributing to lesser fear. Findings from the Hadramout governorate suggest that FOC has a strong cultural component. Maternal identity is strongly influenced by the prevailing familial and sociocultural context. Sense of coherence (SOC) scores and level of FOC have been found to closely correlate (Takegata *et al*. (2014). In their research among Mexican-American women, Gress-Smith *et al*. (2013) identified underlying factors of prenatal expectations: paternal support, family support, and maternal role fulfillment. The viewpoint that childbirth is of God's will and children a gift from God reduced fear among Yemeni women who primarily identified with childbearing as fulfilling an obligation vis-á-vis the husband, kin and society.

### FOC in a South–North perspective

Our study demonstrates the feasibility of using qualitative methodology to detect and uncover those underlying factors in women's lives, which influence their wellbeing and mental health during pregnancy and childbirth. Such factors are likely to also enable or prevent existing childbirth services to be used, both in low- and high-income countries.

In a global framing, findings from Yemen point to the possibility that situations where women are discriminated against become intertwined with FOC. Women's subordinated status in the wider socio-cultural context was shown to increase FOC and vice versa, the strong status of women in one governorate was shown to decrease it. This adds yet another dimension to existing knowledge in the North concerning FOC and women's previous experience of abuse in intimate relationships as well as with staff, the latter of which is being extensively debated. Findings from Yemen could have implications for marginalized groups within other societies.

This work on women's FOC in Yemen is likely to be relevant to policy in other low- and middle-income countries where several systems of health care operate and no efforts have been made of integration. In many low-income countries women are the stakeholders of traditional health care. Where women have a low status findings are likely to apply the strongest.

### Conclusion and implications for practice

Yemeni women's FOC should be seen in the context of personal, socio-cultural and economic factors affecting women, their families and communities. Areas of concern link directly to the maternal MDG 5 and indirectly to MDG 2 concerning universal primary education for boys and girls, MDG 3 about gender equality and MDG 4, which aims to reduce child mortality. It is likely that girls’ education will lessen FOC as girls and young women become aware of their reproductive and human rights. Increasing the legal age for marriage will lessen FOC as women become more equal with their spouses. Decreasing the number of pregnancies and increasing the spacing of pregnancies means that more infants survive, which will lessen FOC. Infrastructural development will lessen FOC as access to professional care during pregnancy and childbirth improves.

What this study shows, above all, is that women's fear needs to be addressed here and now. A reduction of the psychological distress during this important period in a Yemeni woman's life would improve the health and wellbeing not only of new mothers but also their children and families. Actions within MCH care can lessen FOC. ANC has a crucial role in reducing fear including that of institutional childbirth. Finding culturally appropriate arguments to strengthen parents in accepting the equal value of the female infant is important, as is also counseling of women and the provision of childbirth support. Within the scope of strengthening reproductive mental health programs globally, intervention to reduce FOC in Yemen and similar settings is needed.

## A Yemeni girl's plea against early childhood marriage (video)

Yemeni 11-year old Nada al-Ahdal from Hodeidah speaks to the early circumstances in the lives of many Yemeni girls and the effects that early marriage has on their wellbeing and mental health. Provision by courtesy of the Middle East Media Research Institute. http://www.memri.org/report/en/0/0/0/0/0/0/7298.htm
